# Dennd2c Negatively Controls Multinucleation and Differentiation in Osteoclasts by Regulating Actin Polymerization and Protrusion Formation

**DOI:** 10.3390/ijms252111479

**Published:** 2024-10-25

**Authors:** Yu Koyanagi, Eiko Sakai, Yu Yamaguchi, Fatima Farhana, Yohsuke Taira, Kuniaki Okamoto, Hiroshi Murata, Takayuki Tsukuba

**Affiliations:** 1 Department of Dental Pharmacology, Graduate School of Biomedical Sciences, Nagasaki University, Nagasaki 852-8588, Japan; bb55321204@ms.nagasaki-u.ac.jp (Y.K.); eiko-s@nagasaki-u.ac.jp (E.S.); yu-y@nagasaki-u.ac.jp (Y.Y.); fatimafarhana.2011@gmail.com (F.F.); 2 Division of Cariology and Restorative Dentistry, Department of Prosthetic Dentistry, Graduate School of Biomedical Sciences, Nagasaki University, Nagasaki 852-8588, Japan; yohsuke@nagasaki-u.ac.jp (Y.T.); hmurata@nagasaki-u.ac.jp (H.M.); 3 Department of Dental Pharmacology, Graduate School of Medicine, Dentistry and Pharmaceutical Sciences, Okayama University, Okayama 700-8558, Japan; k-oka@okayama-u.ac.jp

**Keywords:** osteoclast, actin polymerization, protrusion formation, Dennd2c

## Abstract

Osteoclasts are bone-resorbing multinucleated giant cells formed by the fusion of monocyte/macrophage lineages. Various small GTPases are involved in the multinucleation and differentiation of osteoclasts. However, the roles of small GTPases regulatory molecules in osteoclast differentiation remain unclear. In the present study, we examined the role of Dennd2c, a putative guanine nucleotide exchange factor for Rab GTPases, in osteoclast differentiation. Knockdown of *Dennd2c* promoted osteoclast differentiation, resorption, and expression of osteoclast markers. Morphologically, *Dennd2c* knockdown induced the formation of larger osteoclasts with several protrusions. In contrast, overexpression of *Dennd2c* inhibited the multinucleation and differentiation of osteoclasts, bone resorption, and the expression of osteoclast markers. *Dennd2c*-overexpressing macrophages exhibited spindle-shaped mononuclear cells and long thin protrusions. Treatment of *Dennd2c*-overexpressing cells with the Cdc42 inhibitor ML-141 or the Rac1 inhibitor 6-thio-GTP prevented protrusion formation. Moreover, treatment of *Dennd2c*-overexpressing cells with the actin polymerization inhibitor latrunculin B restored multinucleated and TRAP-positive osteoclast formation. These results indicate that Dennd2c negatively regulates osteoclast differentiation and multinucleation by modulating protrusion formation in macrophages.

## 1. Introduction

Osteoclasts are predominantly bone-resorbing multinucleated giant cells formed by fusion with macrophages [1]. Osteoclast differentiation is triggered by receptor activator of nuclear factor kappa-B ligand (RANKL) in macrophages. The interaction between RANKL and RANK involves sequential differentiation events, such as cell–cell fusion, cytoskeletal rearrangement, and podosome formation [2]. Various small GTPases modulate these events. The Rho family of GTPases, such as RhoA, Rac1, Cdc42, and RhoU, control cell migration, morphology, motility, and cytoskeletal organization [3,4]. The Rab family of GTPases regulates membrane trafficking, organelle formation, and bone resorption, leading to osteoclast multinucleation and differentiation [5,6]. For example, osteoclasts derived from Rab3D-deficient mice display an abnormal ruffled border membrane and impaired bone resorption but normal actin ring and podosome formation [7,8]. Rab11A and Rab11B negatively regulate osteoclast differentiation. Knockdown of *Rab11A* or *Rab11B* enhances osteoclast differentiation, whereas overexpression of Rab11A or Rab11B impairs it [9,10]. Rab27A-defect osteoclasts derived from *Rab27* gene-mutant ashen mice exhibit abnormal transport of cell surface receptors, enhanced multinucleation, and reduced bone resorption [11]. Rab38 and its paralog Rab32 are involved in the biogenesis of lysosome-related organelles in osteoclasts. *Rab38* and *Rab32* double-knockout osteoclasts show reduced bone resorption activity owing to defective secretion of lysosomal enzymes [12,13]. Although Rab34 and Rab44 differ in their molecular weights, they exhibit similar effects on osteoclast differentiation. Knockdown of *Rab34* or *Rab44* promotes multinucleation differentiation and bone resorption in osteoclasts or overexpression of either gene [14,15]. However, little information is available on the Rab GTPase effectors.

Differentially expressed in normal and neoplastic cells (DENN) domain proteins were originally identified as GDP/GTP exchange proteins for Rab GTPases by biochemical purification from the bovine brain [16]. Generally, DENN proteins function as guanine nucleotide exchange factors (GEFs) for Rab GTPases [17,18]. The human genome contains 18 genes that encode DENN domain proteins, which can be classified into eight families: DENND1A-1C, DENND2A-2D, DENN3, DENND4A-4C, DENND5A/5B, DENND6A/6B, MTMR5/13, and DENN/MADD [19,20]. However, only a limited number of studies have been conducted on *Dennd2c*. Previous genetic studies have shown that single-nucleotide polymorphisms in *Dennd2c* are likely involved in rotator cuff disease, characterized by damaged tendons and/or muscles in the shoulder joint [21,22]. Moreover, a gain-of-function screening of human embryonic stem cells identified Dennd2c, which negatively regulates Rho A activity [23]. Screening experiments using in vitro binding assays for Rab GTPases and DENN domain proteins revealed that Dennd2c interacts with Rab8A, Rab8B, Rab10, Ran15, and Rab35 [24]. However, the physiological function of Dennd2c in these cells remains unclear.

This study investigated the role of Dennd2c in osteoclast differentiation by knockdown and overexpression experiments. We hypothesized that Dennd2c regulate multinucleation and differentiation of osteoclasts.

## 2. Results

### 2.1. Expression of Dennd2c Alters During Osteoclast Differentiation

**Figure 1:**
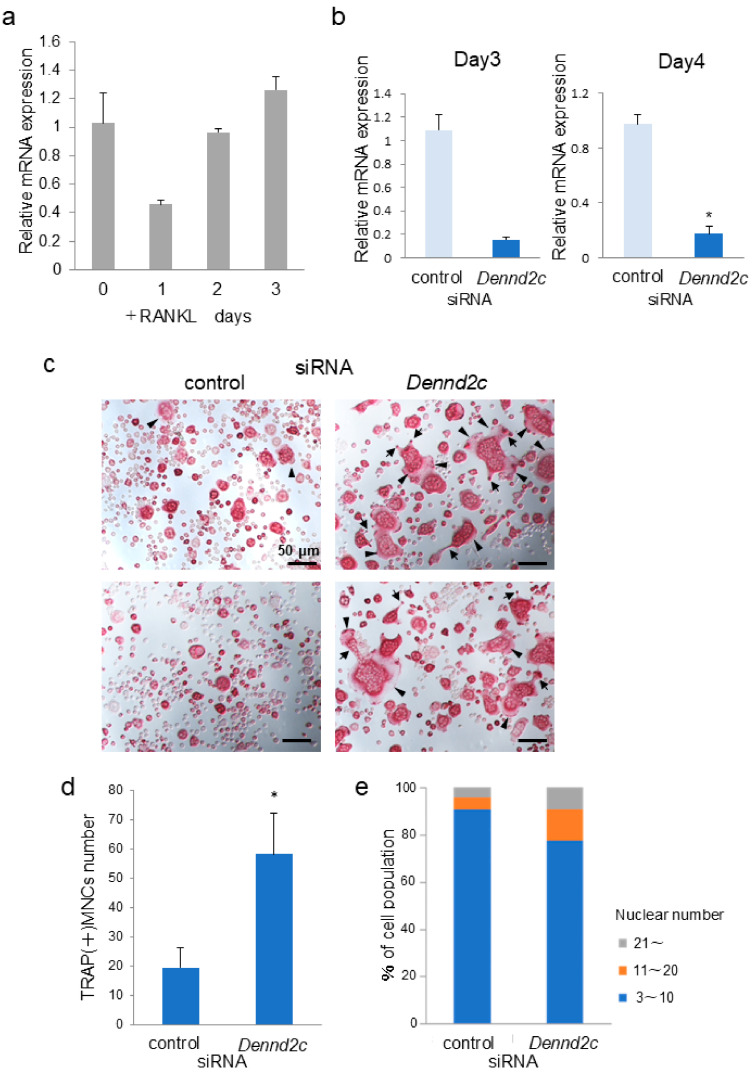
Knockdown of *Dennd2c* promoted osteoclast formation and multinucleation. (**a**) The mRNA expression of native *Dennd2c* in RANKL-stimulated RAW-D cells was measured by real-time PCR. (**b**) The knockdown efficiency of *Dennd2c* was evaluated by measuring the mRNA levels. * p < 0.05, compared with the control cells. (**c**) TRAP staining of control and *Dennd2c*-knockdown osteoclasts. Cells exhibited protrusions (arrows) and lamellipodia (arrowheads): scale bar, 50 μm. (**d**) The number of multinucleated cells (MNCs) was counted between control and *Dennd2c*-knockdown cells. * *p* < 0.05, compared with control cells. (**e**) The total number of nuclei in TRAP-positive multinucleated osteoclasts, but not TRAP-negative mononucleated cells following a four-day culture, was counted and classified per viewing field.

To examine whether the expression levels of *Dennd2c* were altered during osteoclastogenesis, we measured the mRNA levels of *Dennd2c* during osteoclast differentiation in the murine monocytic cell line RAW-D after RANKL stimulation. Quantitative real-time polymerase chain reaction (qRT-PCR) analysis revealed that the mRNA expression of *Dennd2c* decreased on day 1 after RANKL ligand stimulation but recovered on days 2 and 3 (Figure 1a).

### 2.2. Knockdown of Dennd2c Enhances Multinucleation and Differentiation in Osteoclasts

To confirm the role of *Dennd2c* in osteoclastogenesis, knockdown experiments were performed using siRNA-transfected RAW-D cells. When the knockdown efficacy of *Dennd2c* in RAW-D macrophages was measured, *Dennd2c* knockdown resulted in an approximately 85% reduction compared to the control siRNA after three days of transfection (Figure 1b). Similarly, *Dennd2c* knockdown maintained an approximately 80% reduction as compared to the control siRNA after four days of transfection. (Figure 1b).

Tartrate-resistant acid phosphatase (TRAP) staining revealed that *Dennd2c* knockdown induced the formation of larger osteoclasts than the control after four days of RANKL stimulation (Figure 1c). Some of the *Dennd2c*-knockdown osteoclasts showed abnormal cell shapes with abundant protrusions (arrowheads) and lamellipodia (arrows) (Figure 1c). The number of TRAP-positive osteoclasts was significantly higher in *Dennd2c*-knockdown osteoclasts than in the control cells (Figure 1d). When the number of nuclei in the osteoclasts was counted, control osteoclasts containing fewer than 10 nuclei accounted for 90% of the total number, while those with 11–20 nuclei accounted for approximately 6% (Figure 1e). However, *Dennd2c*-knockdown osteoclasts containing more than 21 nuclei accounted for 9% of the total, and 11–20 nuclei cells accounted for 13% (Figure 1e). These results indicate that *Dennd2c* knockdown enhances multinucleation and differentiation of osteoclasts.

### 2.3. Dennd2c Knockdown Markedly Increases Marker Gene Expression in Osteoclasts

**Figure 2:**
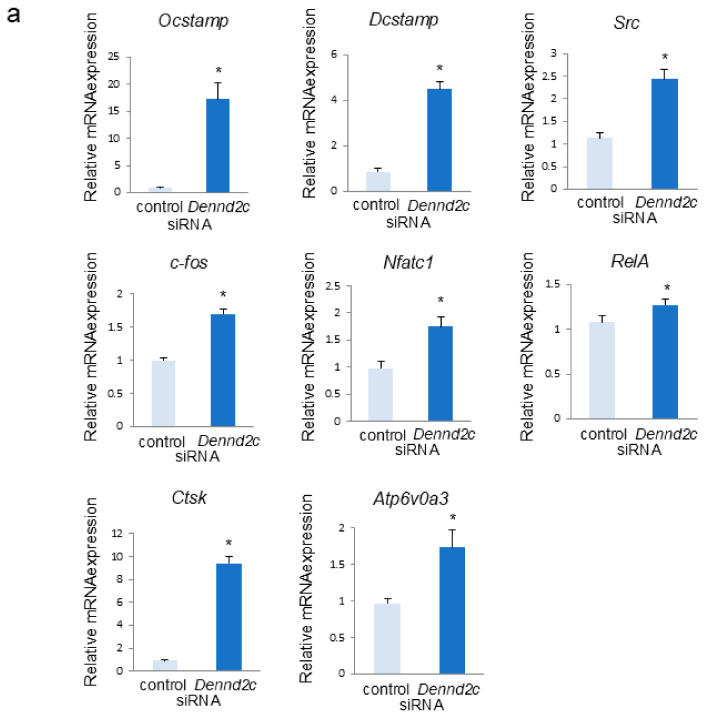

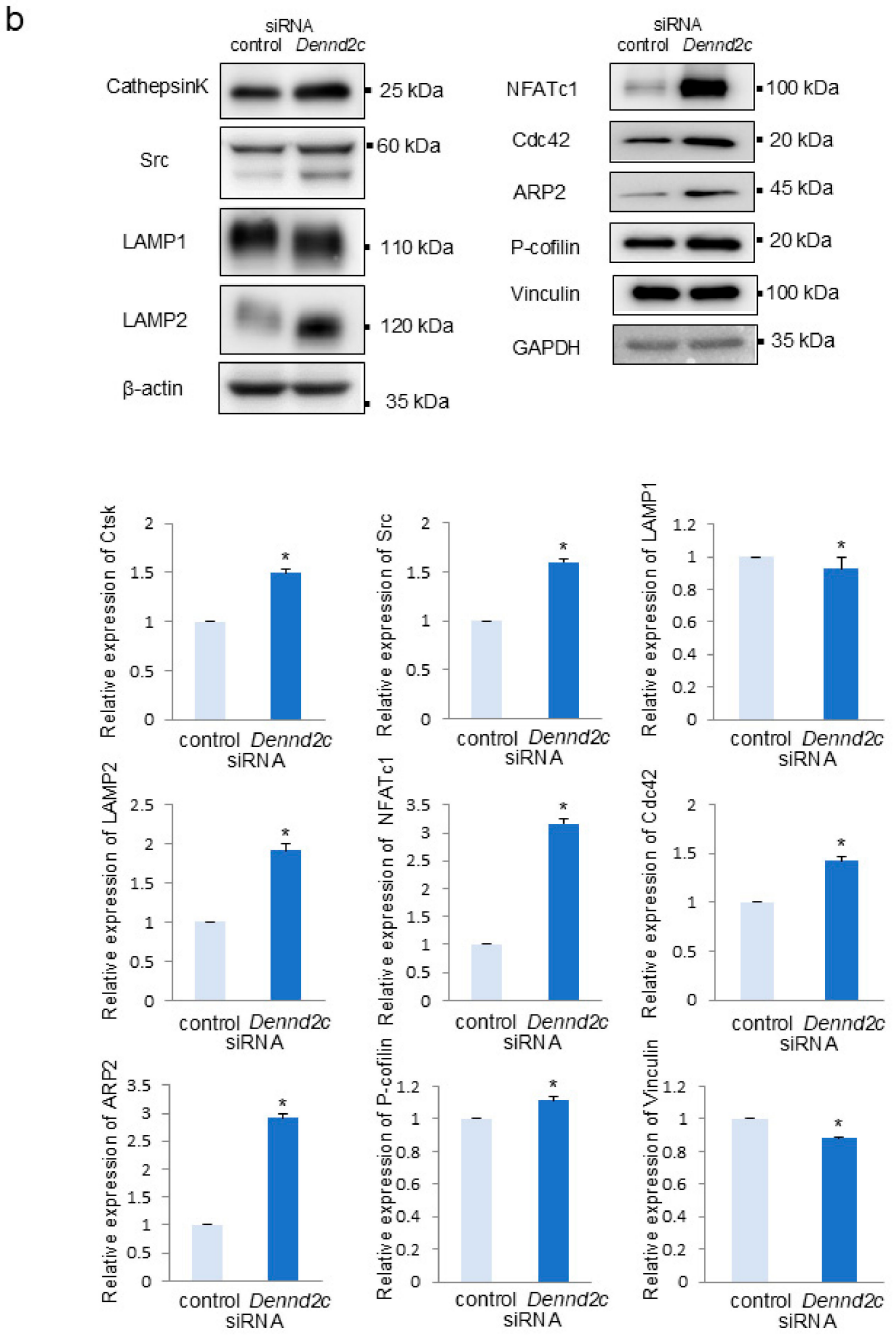
Effects of *Dennd2c* knockdown on osteoclast-marker gene expression. (**a**) The mRNA levels of control and *Dennd2c*-knockdown osteoclasts were measured by real-time PCR. The data are represented as mean ± SD values from three independent experiments. * *p* < 0.05, compared to control cells. (**b**) Western blotting and quantitative analysis of control and *Dennd2c*-knockdown osteoclasts. The data are represented as mean ± SD values from three independent experiments. * *p* < 0.05, compared to control cells.

To evaluate the differences between the control and *Dennd2c*-knockdown osteoclasts, we compared the mRNA levels of various osteoclast marker genes (Figure 2). Marker genes, such as *Nfatc1*, *RelA*, *c-fos, Ocstamp*, *Dcstamp,* and *Src*, participate in the differentiation, fusion, and adhesion of osteoclasts. *CtsK* and *Atp6v0a3* participate in bone resorption and lysosomal function. qRT-PCR results showed that the mRNA levels of all marker genes were higher in *Dennd2c*-knockdown osteoclasts than in control osteoclasts (Figure 2a).

To compare the levels of several proteins in both *Dennd2c*-knockdown and control cells, we performed Western blotting (Figure 2b). The expression levels of Src, cathepsin K, and LAMP2 were significantly upregulated (Figure 2b). Notably, the levels of molecular size of LAMP1 and LAMP2 were slightly reduced. Although the expression levels of LAMP1 in *Dennd2c*-knockdown were decreased, the expression levels of LAMP2 in *Dennd2c*-knockdown cells were higher than that in control cells (Figure 2b). Quantitative analysis of the bands between *Dennd2c*-knockdown and control cells is shown in Figure 2b.

### 2.4. Dennd2c Knockdown Enhances the Resorption Activity of Osteoclasts

**Figure 3:**
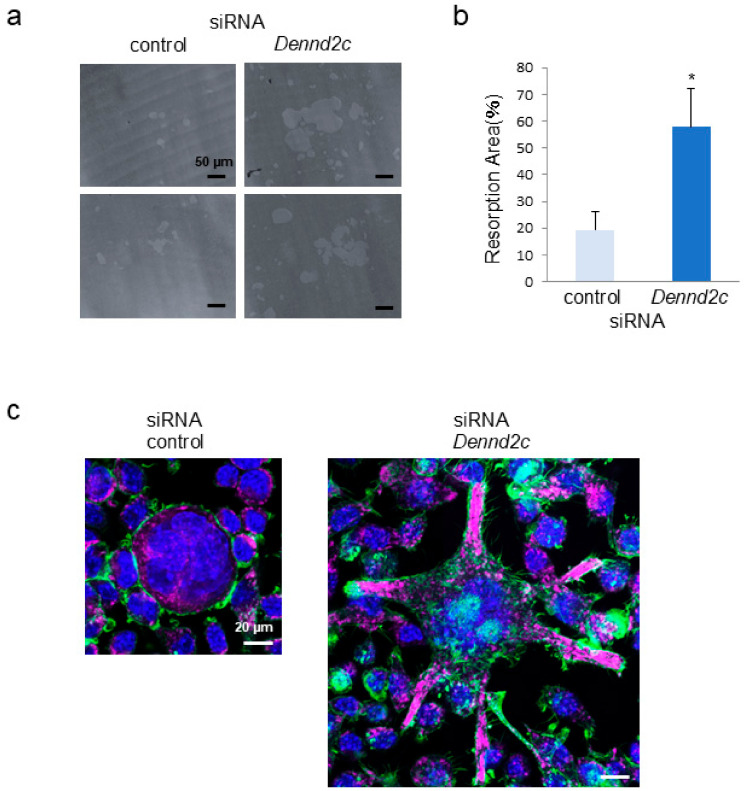
Effects of *Dennd2c* knockdown on bone resorption and morphological features. (**a**) The resorption area of control and *Dennd2c*-knockdown osteoclasts: scale bar, 50 μm. (**b**) The resorption area was measured using ImageJ software (ImageJ 1.54d, National Institutes of Health, Bethesda, MD, USA). The data are shown as mean ± SD values from three independent experiments. * *p* < 0.05, compared with control cells. (**c**) Confocal microscopic analysis of control and *Dennd2c*-knockdown osteoclasts were stained with LAMP2 (magenta), phalloidin (green), and DAPI (blue): scale bars, 20 μm (control and *Dennd2c*-knockdown).

Using a pit formation assay, we examined resorption activities of control and *Dennd2c*-knockdown osteoclasts. Upon determining the resorption areas in the two types of osteoclasts, *Dennd2c*-depleted osteoclasts showed enhanced resorption activity compared with control osteoclasts (Figure 3a). The resorption area calculated using *Dennd2c*-depleted osteoclasts was significantly more extensive than that of the control osteoclasts (Figure 3b). These results indicate that *Dennd2c* depletion results in increased osteoclast-resorbing activity.

### 2.5. Dennd2c Knockdown Induces Larger Osteoclast Formation with Several Protrusions Containing LAMP2-Positive Compartments

We performed an immunofluorescence analysis of *Dennd2c*-knockdown osteoclasts. When we observed the staining patterns of actin and LAMP2 in control and *Dennd2c*-knockdown osteoclasts, the *Dennd2c*-knockdown osteoclasts displayed LAMP2-positive compartments in the several protrusions as well as in the cytoplasm. In contrast, control osteoclasts possessed LAMP2-positive compartments in the cytoplasm (Figure 3c). These results suggest that *Dennd2c* knockdown induces larger osteoclast formation with lysosome-containing protrusions.

### 2.6. Overexpression of Dennd2c Inhibits the Multinucleated Formation and Differentiation in Osteoclasts

**Figure 4:**
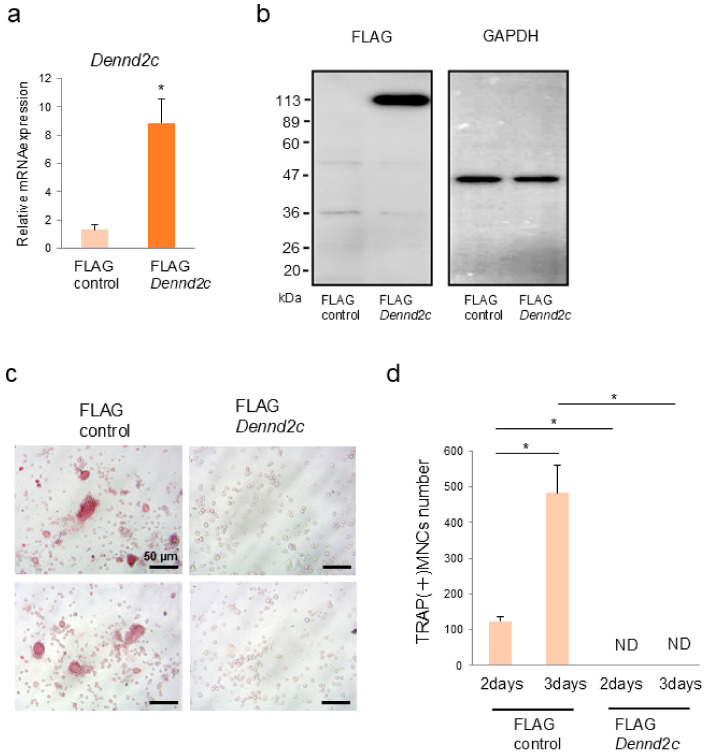
Overexpression of *Dennd2c* inhibited osteoclast formation, resorption area, and osteoclast-marker gene expression. (**a**) mRNA levels in control and Dennd2c-overexpressing osteoclasts were measured by real-time PCR. The data are represented as mean ± SD values from three independent experiments. * *p* < 0.05, compared with control cells. (**b**) Western blotting of control and *Dennd2c*-overexpressing osteoclasts. (**c**) TRAP staining of control and *Dennd2c*-overexpressing osteoclasts: scale bar, 20 μm. (**d**) The number of TRAP-positive multinucleated cells (MNCs) in the control and *Dennd2c*-overexpressing cells counted on the indicated day. * *p* < 0.05, compared with the control cells.

Next, we conducted overexpression experiments in RAW-D cells using FLAG-tagged vectors encoding FLAG–*Dennd2c*, or FLAG only. qRT-PCR analysis revealed that the mRNA level in *FLAG–Dennd2c*-expressing cells was approximately 9-fold higher than that in control cells (Figure 4a). Western blotting using an anti-FLAG antibody indicated that the FLAG–Dennd2c protein was detectable as a major band of approximately 115 kDa in the overexpressing cells (Figure 4b). TRAP staining was performed to determine whether *Dennd2c* overexpression affected osteoclast differentiation. After 3 days of RANKL stimulation, *Dennd2c* overexpression almost completely prevented TRAP-positive multinucleated osteoclast differentiation compared to that in control cells (Figure 4c). In addition, the number of multinucleated cells in *Dennd2c*-overexpressing cells was reduced entirely compared to that in control osteoclasts (Figure 4d). The resorption activity of control and *Dennd2c*-overexpressing osteoclasts was examined. As expected, *Dennd2c*-overexpressing osteoclasts completely abolished resorption activity. These results indicate that *Dennd2c* overexpression inhibits multinucleated osteoclast formation and differentiation.

### 2.7. Dennd2c Overexpression Markedly Reduces Marker Gene Expression in Osteoclasts

**Figure 5:**
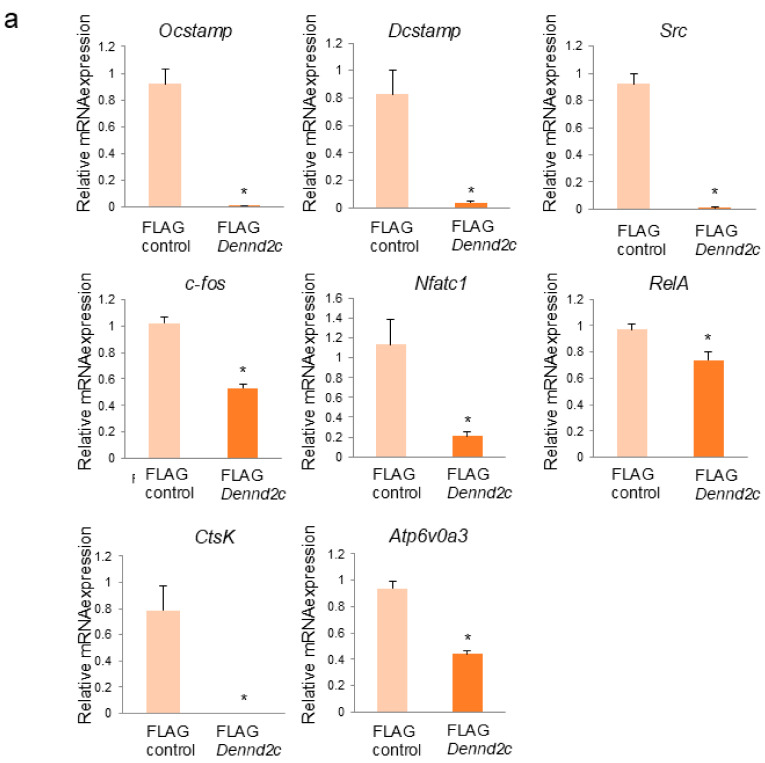

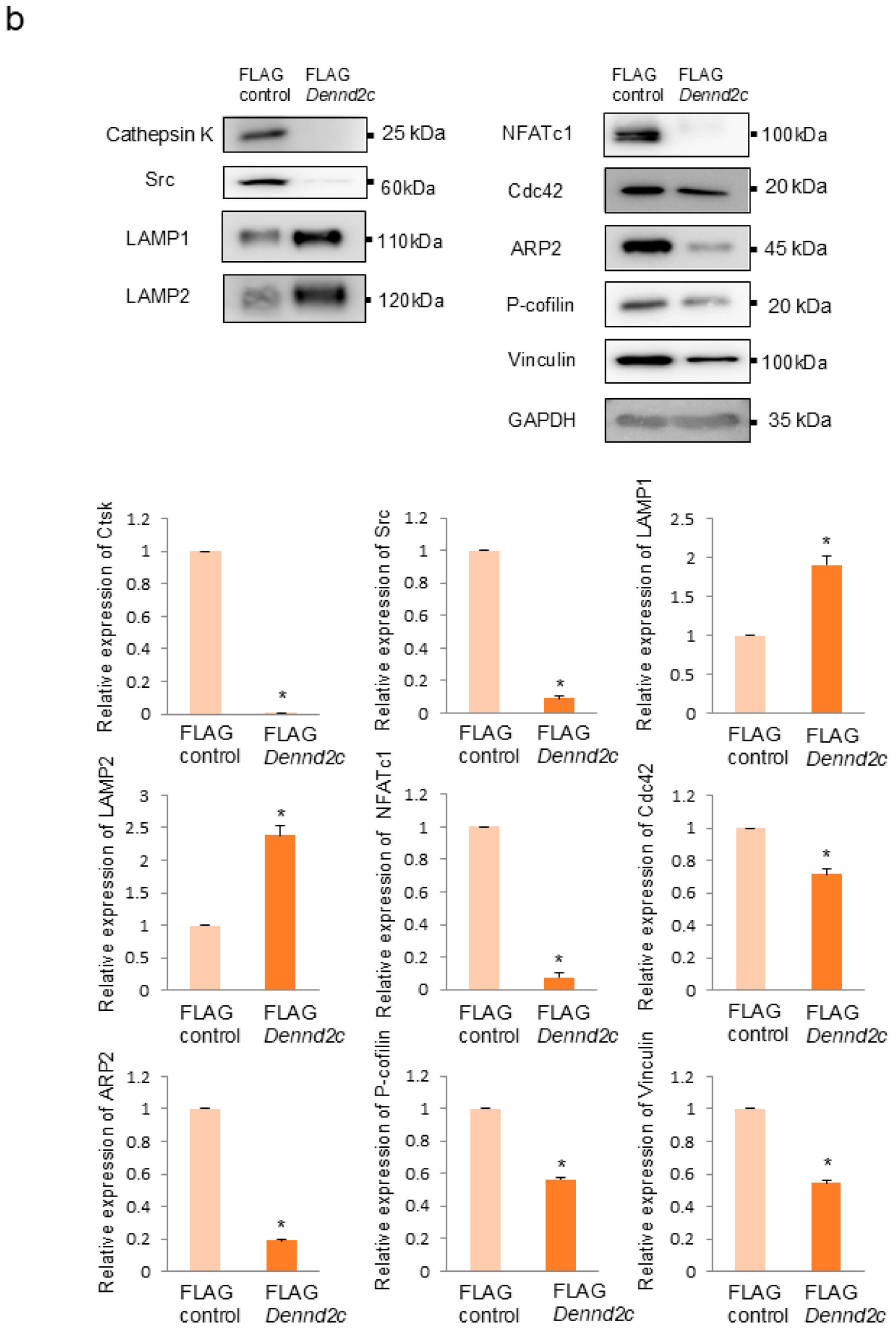
Effects of *Dennd2c* overexpression on osteoclast-marker gene expression. (**a**) The mRNA levels of control and *Dennd2c*-overexpressing osteoclasts were measured by real-time PCR. The data are represented as mean ± SD values from three independent experiments. * *p* < 0.05, compared to control cells. (**b**) Western blotting and quantitative analysis of control and *Dennd2c*-overexpressing osteoclasts. The data are represented as mean ± SD values from three independent experiments. * *p* < 0.05, compared to control cells.

We also examined the gene and protein expression of osteoclast-related markers in the control and *Dennd2c*-overexpressing osteoclasts. qRT-PCR was performed for several osteoclast markers. In *Dennd2c*-overexpressing osteoclasts, all markers were significantly downregulated compared to those in the control osteoclasts (Figure 5a). Western blotting indicated that NFATc1, Src, and cathepsin K levels were hardly detectable in *Dennd2c*-overexpressing osteoclasts compared to those in control osteoclasts (Figure 5b). Furthermore, the levels of vinculin, Cdc42, ARP2, and P-cofilin were significantly decreased, but those of LAMP1 and LAMP2 were significantly increased in *Dennd2c*-overexpressing osteoclasts compared with those in the control cells (Figure 5b). Quantitative analysis of the bands between *Dennd2c*-overexpressing and control cells is shown in Figure 5b. These results indicate that *Dennd2c*-overexpressing downregulates osteoclast markers and cytoskeleton/adhesion molecules but upregulates lysosomal membranes.

### 2.8. Dennd2c-Overexpressing Cells Form Spindle-Shape and Long and Thin Protrusions

**Figure 6:**
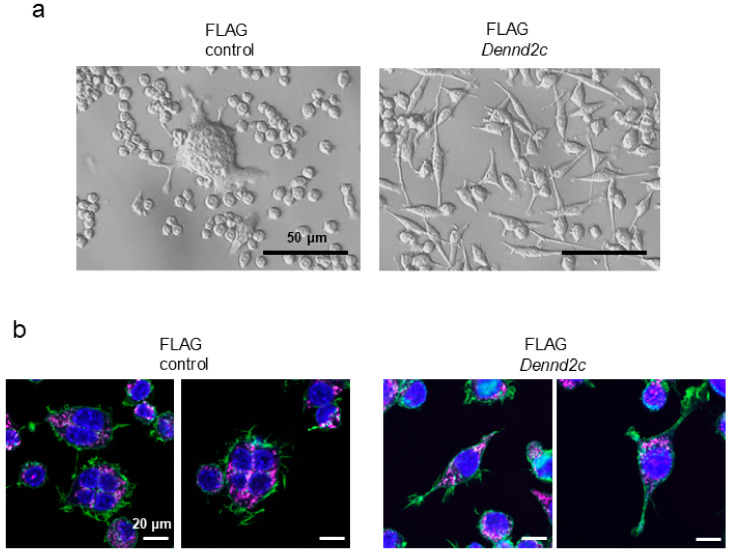
*Dennd2c*-overexpression formed spindle-shaped and abnormal protrusions. (**a**) Phase-contrast microscopic observation of control and *Dennd2c-*overexpressing osteoclasts: scale bar, 50 μm. (**b**) Confocal microscopic analysis of control and *Dennd2c*-overexpressing osteoclasts stained with LAMP2 (magenta), phalloidin (green), and DAPI (blue). Scale bars, 20 μm.

A series of morphological observations using phase-contrast microscopy revealed that *Dennd2c*-overexpressing cells were spindle-shaped and formed abnormal protrusions (Figure 6a). In contrast, control cells formed multinucleated cells and partially fused round cells (Figure 6a). When we observed the morphological characteristics of the control and *Dennd2c*-overexpressing osteoclasts using immunofluorescence staining, the control osteoclasts displayed LAMP2-positive dots in the cytoplasm and were surrounded by actin/phalloidin-positive reactivity at the cell edge (Figure 6b). However, *Dennd2c*-overexpressing osteoclasts exhibited LAMP2-positive lysosomes in the cytoplasm and actin/phalloidin-positive reactivity in long and thin protrusions (Figure 6b). In the protrusions of *Dennd2c*-overexpressing osteoclasts, however, LAMP2 positive immunoreactivity was not observed, suggesting that the protrusions are not likely to be required for secretion like neurites but rather for cell–cell adhesion or motility (Figure 6b). Moreover, overexpressing cells tended to detach easily.

### 2.9. Effects of Cytoskeleton-Related Inhibitors on the Multinucleation and Protrusion Formation of Dennd2c-Overexpressing Cells

**Figure 7:**
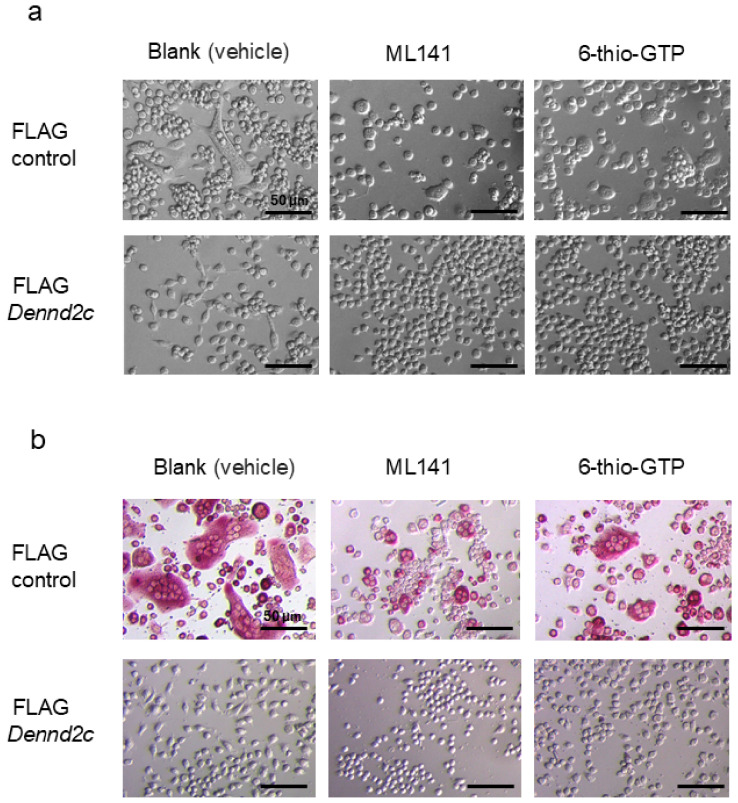
Effects of ML141 and 6-thio-GPT on the multinucleation and protrusion formation of *Dennd2c*-overexpressing cells. (**a**,**b**) Control and *Dennd2c*-overexpressing osteoclasts in the presence or absence of 10 μM L-141 or 10 μM 6-thio-GTP. Cells without staining were observed using phase-contrast microscopy (**a**) or TRAP staining using optical microscopy (**b**): scale bar, 50 μm.

To examine the mechanisms underlying protrusion formation, we treated control and *Dennd2c*-overexpressing cells with the Cdc42 inhibitor ML-141 or the Rac1 inhibitor 6-thio-GTP (Figure 7a). Both ML-141 and 6-thio-GTP inhibited protrusions in *Dennd2c*-overexpressing cells (Figure 7a). However, these inhibitors did not ameliorate multinucleation and differentiation (Figure 7b). Moreover, these inhibitors did not restore TRAP-positive (Figure 7b). These results indicate that *Dennd2c* overexpression induces abnormal actin polymerization, which prevents multinucleation, differentiation, and abnormal protrusion formation regulated by Cdc42 and/or Rac1.

**Figure 8:**
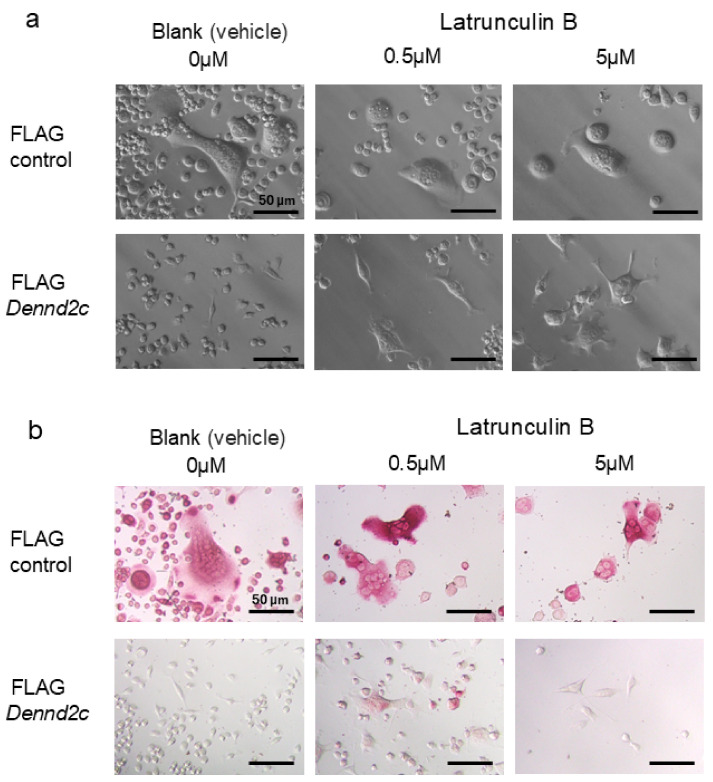
Effects of latrunculin B on the multinucleation and protrusion formation of *Dennd2c*-overexpressing cells. (**a**,**b**) Control and *Dennd2c*-overexpressing osteoclasts in the presence or absence of 0.5 or 5 μM latrunculin B. Cells were observed without staining using phase-contrast microscopy (**a**) or with TRAP staining using optical microscopy (**b**): scale bar, 50 μm.

We also treated the cells with latrunculin B, an actin polymerization inhibitor (Figure 8a). Interestingly, *Dennd2c*-overexpressing cells treated with latrunculin B showed increased multinucleation, a decreased number of spindle-shaped cells, and protrusions with lamellipodia-like structures at their tips (Figure 8a). TRAP staining revealed that 0.5 μM of latrunclin B-treated *Dennd2c*-overexpressing cells were changed into partially TRAP-positive (Figure 8b). *Dennd2c*-overexpressing cells appeared to detach easily during TRAP staining. When 5 μM of latrunculin B was used, a significant portion of the cells were lost, and no TRAP-positive cells were discernible. These results indicate that latrunculin B suppresses actin polymerization in the long-axis direction, thereby promoting the formation of protrusions with lamellipodia-like structures and osteoclast differentiation, suggesting that Dennd2c-mediated actin polymerization in the long-axis direction impairs osteoclast differentiation.

## 3. Discussion

In this study, we demonstrated that *Dennd2c* knockdown enhanced osteoclastic multinucleation, increased resorption activity, and elevated the expression of several osteoclast markers. Conversely, *Dennd2c* overexpression prevented the differentiation and multinucleation of macrophages into osteoclasts and inhibited the expression of osteoclast marker genes. Morphologically, *Dennd2c*-overexpressing macrophages exhibited spindle-shaped mononuclear cells and formed protrusions. Pharmacological analysis revealed that the actin polymerization inhibitor latrunculin B recovered multinucleated and TRAP-positive osteoclast formation, whereas the Cdc42 inhibitor ML-141 or the Rac1 inhibitor 6-thio-GTP prevented protrusion formation. Thus, *Dennd2c* negatively regulates osteoclast differentiation and multinucleation by modulating protrusion formation in macrophages.

*Dennd2c* knockdown enhanced osteoclast differentiation and multinucleation, whereas *Dennd2c* overexpression reversed these effects. *Dennd2c* is likely involved in the differentiation of cells other than osteoclasts. A previous study on human embryonic stem cells reported that *DENND2C* overexpression blocks retinoic acid (RA)-induced differentiation [23]. In that study, *DENND2C* genetically cooperates with NANOG, a transcription factor for pluripotency, to maintain self-renewal [23]. However, *DENND2C* knockdown had no effect on RA-induced differentiation. Thus, *Dennd2c* is likely involved in the differentiation of osteoclasts and embryonic stem cells, although the effects of knockdown are different between macrophages and stem cells.

Alterations in Dennd2c expression likely caused morphological changes. Although *Dennd2c* knockdown osteoclasts exhibited abnormal structures, *Dennd2c*-overexpressing macrophages exhibited spindle-shaped mononuclear cells and formed protrusions. Consistent with our findings, *Dennd2c*-overexpressing embryonic stem cells display a strikingly disorganized cortical F-actin staining pattern [23]. DENND2-mediated morphological changes are associated with functional changes. This hypothesis is supported by the finding that *Dennd2c*-overexpressing cells exhibit weak adhesion and reduced bone resorption. Similarly, *Dennd2c*-overexpressing stem cells have a weak attachment to Matrigel and easily detached after collagenase IV treatment [23].

However, the detailed mechanisms by which alterations in *Dennd2c*-overexpressing cells cause morphological changes remain unclear. One possible explanation is that Dennd2c is directly involved in Rho GTPases such as RhoA, Cdc42, and Rac1. In this study, treatment of *Dennd2c*-overexpressing cells with the Cdc42 inhibitor ML-141 or the Rac1 inhibitor 6-thio-GTP prevented protrusion formation. Similarly, *DENND2C*-overexpressing embryonic stem cells displayed nuclear translocation of RhoA, whereas control cells showed cytoplasmic localization of RhoA [23]. However, in that study, co-immunoprecipitation of Dennd2c did not detect a direct interaction between Dennd2c and RhoA or RAC1 [23]. Considering that some DENN-related proteins have been reported to act as effectors of small GTPases in addition to Rab proteins, Dennd2c may also function as an effector of Cdc42 and Rac1.

Another situation of involvement of Dennd2c in morphological changes is that Dennd2c is indirectly associated with Cdc42/Rac1 GTPases through direct interaction with Rab35, which regulates F-actin rearrangement. Previous in vitro screening of binding experiments using HeLa cells showed that Dennd2c interacts with several Rab proteins, including Rab8A, Rab8B, Rab9, Rab10, Rab15, and Rab35 [24]. Rab35 is a key regulator of F-actin polymerization through Rac1/Cdc42 activation [25]. Interestingly, the phenotypes of *Dennd2c*-overexpressing macrophages and Rab35-overexpressing neuronal cells with extended membrane protrusions are similar. Rab35 colocalizes with Cdc42, Rac1, and RhoA in various neuronal cells, activates Cdc42, and stimulates neurite outgrowth in a Cdc42-dependent manner through actin remodeling [26]. Moreover, the constitutively active mutant Rab35Q67L enhances neurite outgrowth in neuronal cells, whereas the dominant negative mutant Rab35S22N completely inhibits this extension [26]. *Dennd2c*-overexpressing cells exhibited spindle-shaped mononuclear cells and formed protrusions, suggesting that Dennd2c cooperates with Rab35, Cdc42, Rac1, and RhoA to regulate cytoskeletal organization and bone resorption via actin polymerization in osteoclasts.

Finally, we discuss the medical applications of the findings of this study. Several genetic studies have reported that gene mutations in *Dennd2c* are implicated in rotator cuff disease, which is characterized by damaged tendons and muscles in the shoulder joint [21,22]. Calcium deposition and the subsequent process of absorption in damaged tendons can cause severe pain. Nakase et al. [27] previously showed that cathepsin K-positive osteoclast-like cells are involved in the resorption of calcium deposits in this disease. Given that the *Dennd2c* mutation results in abnormal resorption of calcium deposits in rotator cuff disease, our findings may provide clues to the mechanisms, diagnostics and therapeutic approaches for this disease. To further explore the function of *Dennd2c* in vivo, analyses using transgenic or conditional knockout mice would be helpful.

## 4. Materials and Methods

### 4.1. Reagents

Fetal bovine serum (FBS) was purchased from Sigma-Aldrich (St. Louis, MO, USA). The minimum essential medium (MEM) alpha medium (α-MEM) was purchased from WAKO (Osaka, Japan). Recombinant RANKL was prepared as previously described [28]. Antibodies used for Western blot analysis are as follows: anti-GAPDH (catalogue number [Cat. no.] Cat. no. 5174); anti-vinculin (Cat. no. 13901); anti-Arp2 (Cat. no. 5614); anti-phospho-cofilin (Cat. no. 3313); anti-Cdc42 (Cat. no. 2462); anti-mouse IgG, HRP-linked antibody (Cat. no. 7076); anti-rabbit IgG, HRP-linked antibody (Cat. no. 7074); Anti-rat IgG (H+L)-Alexa 555 conjugate (Cat. no. 4417) antibody; horseradish peroxidase-conjugated secondary antibodies were purchased from Cell Signaling Technology (Danvers, MA, USA). Anti-Src antibody (Cat. no. 05-184, mouse monoclonal) was purchased from Merck Millipore (Darmstadt, Germany). Anti-NFATc1 antibody (Cat. no. SC-7294) was purchased from Santa Cruz (Dallas, TX, USA). Anti β-actin antibody (Cat. no. A-5060) and anti-FLAG antibody (Cat. no. F-1804, clone M2) were purchased from Sigma-Aldrich (St. Louis, MO, USA). Alexa Fluor 488 phalloidin (Cat. no. A12379) and ProLong Diamond Antifade Mountant with DAPI (Cat. no. p36962) were purchased from Invitrogen (Carlsbad, CA, USA). Anti-LAMP1 (CD107a, lysosome-associated membrane protein-1, rat monoclonal) and Anti-LAMP2 (CD107b, lysosome-associated membrane protein-2, rat monoclonal) antibodies were prepared using hybridoma cells kindly provided by Dr. Miki Yokoyama (Tokyo Medical and Dental University, Tokyo, Japan). Briefly, hybridoma cell culture supernatants of 1D4B (LAMP1) or H4B4 (LAMP2) were collected, and the anti-LAMP1 IgG or anti-LAMP2 IgG in the supernatant were eluted using Protein G-Sepharose. The presence of the IgG fraction was then confirmed by electrophoresis. The anti-cathepsin K antibody was prepared as previously described [15]. Osteo Assay Plates were purchased from Corning (Corning, NY, USA). Phenylmethylsulfonyl fluoride (PMSF) and a protease inhibitor cocktail (Cat. no. P8340) were purchased from Sigma-Aldrich (St. Louis, MO, United States). Latrunculin B (Cat. no. AG-CN2-0031) was purchased from AdipoGen Life Sciences (San Diego, CA, USA). ML141 (Cat. no. S7686) was purchased from Selleck Chem (Houston, TX, USA). 6-Thio-GTP (Cat. no. ab146746) was purchased from Abcam (Cambridge, UK).

### 4.2. Cell Culture

RAW-D, a murine macrophage cell line, was kindly provided by Prof. Toshio Kukita (Kyushu University, Fukuoka, Japan); the cells were cultured as reported previously [29,30]. RAW-D, a subclone of RAW264 cells, is a mouse osteoclast precursor cell line that efficiently differentiates into multinucleated osteoclasts upon after RANKL stimulation. RAW-D cells were incubated in α-MEM containing 10% FBS and 1% penicillin-streptomycin in 100 mm dishes at 37 °C and in a 5% CO_2_ atmosphere. After reaching 70% confluence, the cells were collected by gentle pipetting without scraping and used in different experiments.

### 4.3. Retrovirus Construction and Overexpression of Dennd2c

Mouse *Dennd2c* complementary DNA (cDNA) was amplified by PCR using specific primers and cDNA derived from RAW-D cells was incubated with RANKL for 72 h as a template. Briefly, cDNA encoding the full-length mouse *Dennd2c* was amplified by PCR using PrimeSTAR GXL DNA Polymerase (Takara, Tokyo). The primers used for PCR were as follows: forward (1xFLAG–*Dennd2c*, forward primer):5′-GACGACGATGATAAGCACCCCACCGGGAACAT-3′ and reverse (vector-EcoRI-*Dennd2c*, reverse primer): 5′-TCCCCTACCCGGTAGAATTCTCACTTCTTGTGAAGAAATTTCATTTTACTTCCAAGACT-3′. Retrovirus construction was constructed using an enhanced green fluorescent protein (eGFP)-tagged pMSCVpuro retroviral vector, kindly provided by Prof. Kosei Ito, Nagasaki University, Nagasaki, Japan [15]. Briefly, the eGFP region was eliminated from the eGFP-tagged pMSCVpuro retroviral vector by XhoI and EcoR1. 1xFLAG-tagged pMSCVpuro retroviral vector was constructed with synthetic oligonucleotides: 5′-CCGGAATTAGATCTCTCGAGATGGACTACAAGGACGACGATGATAAGGAATTCTACCGGGTAGGGGAG-3′, a forward primer with an XhoI site (5′-CCGGAATTAGATCTCTCGAGATGG-3′), and a reverse primer with an EcoRI site (5′-CTCCCCTACCCGGTAGAATTC-3′). To produce the 1xFLAG–*Dennd2c* fusion protein, an InFusion reaction was performed using the PCR with described above fragments of *Dennd2c* and the linearized FLAG-tagged pMSCVpuro vector. According to the manufacturer’s instructions, 1xFLAG alone and 1xFLAG–*Dennd2c* vector constructs were transfected into HEK293T cells using Lipofectamine 3000 (Life Technologies, Carlsbad, MD, USA). After culturing at 37 °C in 5% CO_2_ for 48 h, virus-containing supernatants were collected and used to infect RAW-D cells. *Dennd2c*-overexpressing RAW-D cells were selected in α-MEM containing puromycin (5 μg/mL). Subsequently, fresh media were changed every 3 days. Several cloned cells were obtained approximately 2 weeks later.

### 4.4. Gene Knockdown by siRNA

The transfection of RAW-D cells with siRNA was performed as previously described [28]. Synthetic siRNA oligonucleotides specific for *Dennd2c* were designed and synthesized by Invitrogen. (siRNA1: 5′-CCUUCUCGAGCGGAGGGUAAUCUUU-3′, siRNA2: 5′-GGAGACACAGAUGUUUGCAGGAUUU-3′, and siRNA3: 5′-CCAAAGAAAUAUGGCGGGAAGAUCA-3′). RAW-D cells were transfected with siRNAs (10 nM/transfection) using Lipofectamine RNAiMAX (Invitrogen). After 24 h of transfection, the cells were transfected with the siRNA and incubated for 24 or 48 h. Stealth siRNA Negative Control Duplexes (Invitrogen) were used as negative controls.

### 4.5. Osteoclast Differentiation and Tartrate-Resistant Acid Phosphatase (TRAP) Staining

To induce osteoclast differentiation, *Dennd2c*–siRNA-treated and control siRNA-treated RAW-D cells, as well as *Dennd2c*–FLAG-overexpressing RAW-D and control cells, were cultured in a complete medium containing 100 ng/mL RANKL for three or four days. The cells were then fixed with 4% paraformaldehyde (PFA) in phosphate-buffered saline (PBS) for 30 min on ice and permeabilized with 0.2% Triton X-100 in PBS at 25 °C for 5 min. Finally, the cells were incubated with 0.01% naphthol AS-MX phosphate (Sigma-Aldrich) and 0.05% fast red violet LB salt (Sigma-Aldrich) in the presence of 50 mM sodium tartrate and 90 mM sodium acetate (pH 5.0) to measure the TRAP activity. TRAP-positive cells with three or more nuclei were considered mature osteoclasts.

### 4.6. Western Blotting and Denstmetric Analysis

Cells were stimulated with RANKL (100 ng/mL) for the indicated times. The cells were then rinsed twice with ice-cold PBS and lysed in cell lysis buffer (50 mM Tris-HCl [pH 8.0], 1% Nonidet P-40, 0.5% sodium deoxycholate, 0.1% sodium dodecyl sulfate [SDS], 150 mM NaCl, 1 mM PMSF, and proteinase inhibitor cocktail). The indicated protein amounts (5 µg) were subjected to SDS-polyacrylamide gel electrophoresis (SDS-PAGE), and then transferred onto a polyvinylidene difluoride membrane. The blots were blocked with 3% skim milk in Tris-buffered saline for 1 h at 25 °C, incubated with primary antibody of target protein at 4 °C overnight, washed, incubated with horseradish peroxidase-conjugated secondary antibodies, and finally detected with Immobilon Forte Western HRP substrate (Merck, Darmstadt, Germany). Immunoreactive bands were analyzed using a LAS-4000 mini (Fujifilm, Tokyo, Japan). The digital data of the optical density was quantified using Image J software.

### 4.7. Quantitative (q) Real-Time PCR (qRT-PCR) Analysis

qRT-PCR was performed as previously described [15]. Total RNA was extracted using TRIzol Reagent (Invitrogen). Reverse transcription was performed using oligo(dT)15 primer (Promega, Madison, WI, USA) and ReverTra Ace (Toyobo, Osaka, Japan). The qRT-PCR was performed using Quant Studio 3 (Thermo Fisher Scientific, Waltham, MA, USA). cDNA was amplified using the Brilliant III Ultra-Fast SYBR QPCR Master Mix (Agilent Technologies, Santa Clara, CA, USA), according to the manufacturer’s instructions. The following primer sets were used (5′ to 3′):
*Dennd2c*forward: CACCCCACCGGGAACATGGATGTT reverse: TCACTTCTTGTGAAGAAATTTCATTTTACTTC*Atp6v0a3*forward: GCCTCAGGGGAAGGCCAGATCG reverse: GGCCACCTCTTCACTCCGGAA *Ctsk*forward: CAGCTTCCCCAAGATGTGAT reverse: AGCACCAACGAGAGGAGAAA*C-fos*forward: CCA GTC AAG AGC ATC AGC AA reverse: AAG TAG TGC AGC CCG GAG TA*Dcstamp*forward: CTAGCTGGCTGGACTTCATCC reverse: TCATGCTGTCTAGGAGACCTC*Ocstamp*forward: TGGGCCTCCATATGACCTCGAGTAG reverse: TCAAAGGCTTGTAAATTGGAGGAGT*RelA*forward: GCGTACACATTCTGGGGAGT reverse: GTTAATGCTCCTGCGAAAGC*Src*forward: AGAGTGCTGAGCGACCTGTGT reverse: GCAGAGATGCTGCCTT-GGTT

### 4.8. Immunofluorescence Microscopy

Immunofluorescence microscopy was performed as previously described [15]. *Dennd2c*–FLAG-overexpressing RAW-D cells or control cells were stimulated with RANKL (100 ng/mL). After 3 days of treatment, the cells were fixed with 4% PFA in PBS for 20 min, permeabilized with 0.2% Triton X-100 in PBS for 10 min at 25 °C, and blocked with 0.2% gelatin in PBS for 1 h at 25 °C. The cells were incubated overnight with primary antibodies against LAMP1. After washing three times with PBS, the cells were stained with an anti-rat IgG-Alexa Fluor 555 conjugate as a secondary antibody. Alexa Fluor 488 phalloidin was used to visualize F-actin. Cells were mounted and stained by ProLong Diamond Antifade Mountant and DAPI. Samples were analyzed by microscopy using a laser-scanning confocal imaging system (LSM800; Carl Zeiss, AG, Jena, Germany).

### 4.9. Bone Resorption Assay

RAW-D cells were cultured in Osteo Assay Stripwell Plates (Corning) and stimulated with 500 ng/mL of RANKL for 10 days. After incubation of 72 h, half of the culture medium was changed with an equal amount of α-MEM supplemented with RANKL and FBS. After 10 days, the osteoclasts were lysed with 5% sodium hypochlorite. Images of the resorbed pits were determined using a reverse-phase microscope (CKX41, Olympus, Tokyo, Japan). As previously described, the ratios of the resorbed areas to the total areas were calculated using Image J software [16].

### 4.10. Phase Contrast Microscopy

FLAG-tagged *Dennd2c*-overexpressing and FLAG RAW-D cells were stimulated with RANKL (100 ng/mL) in 96-well plates. After three days, morphological images were captured using a reverse-phase microscope (CKX41, Olympus).

### 4.11. Statistical Analysis

All values are expressed as means ± standard deviation (SD) of three independent experiments. The Mann–Whitney U test determined statistical significance by comparing the two groups. Differences were considered statistically significant at * *p* < 0.05.

## 5. Conclusions

The present study, using knockdown and overexpression methods, indicates that *Dennd2c* negatively controls multinucleation and differentiation in osteoclasts. *Dennd2c*-overexpressing macrophages are spindle-shaped mononuclear cells with protrusions. Pharmacological treatment with several inhibitors has revealed that actin polymerization was involved in multinucleated and TRAP-positive osteoclast formation. In contrast, Rac1 regulates protrusion formation. Taken together, *Dennd2c* negatively regulates osteoclast differentiation and multinucleation by modulating protrusion formation in macrophages.

## Data Availability

The authors declare that all the data supporting this study’s findings are available in this article.
